# Long-term variability of bioclimatic conditions and tourism potential for Warsaw agglomeration (Poland)

**DOI:** 10.1007/s00484-020-01957-2

**Published:** 2020-06-30

**Authors:** Katarzyna Rozbicka, Tomasz Rozbicki

**Affiliations:** Department of Hydrology, Meteorology and Water Management, Institute of Environmental Engineering, Warsaw University of Life Sciences WULS-SGGW, Warsaw, Poland

**Keywords:** Bioclimatic condition, Tourism potential, Thermal stress, UTCI index, Climate-tourism-information-scheme (CTIS), Warsaw

## Abstract

The research area includes one of the largest in terms of population and also the most attractive tourist area in Poland—Warsaw agglomeration. The aim of the study is to assess the temporal and spatial difference of the heat stress on the human body in this area based on long-term data (1980–2016). On the basis of the Universal Thermal Climate Index (UTCI) and designated Climate-Tourism-Information-Scheme (CTIS) diagrams, a comprehensive and detailed bioclimate assessment was made for three different areas. The highest values of the UTCI as well as the frequency of thermal sensations related to heat stress occurred at the Bielany station representing the city area, and the lowest at stations representing suburban area—Legionowo and outskirts—Okęcie. A negative linear trend of the number of days was observed for thermal stress related to cold stress and the category of ‘no thermal stress’ while a positive linear trend was obtained for thermal stress related to heat stress. Based on the obtained results, it can be concluded that in summer months (in June over 60%) the conditions are favourable for the residents causing the lack or slight intensification of the body’s adaptation processes and they are beneficial for practicing various forms of recreation and tourism. However, on the other hand, the rise in the number of days with the ‘strong and very strong heat stress’ especially at the station representing the city area is a disturbing factor and negatively affecting both the health and well-being of agglomeration residents in the future.

## Introduction

Both short- and long-term bioclimatic conditions have a significant impact on the quality of life in the urban environment. These conditions are modified due to environmental factors, development and the complex structure of urban agglomerations. Expanded research in the field of human biometeorology indicates the impact of the urban bioclimate on morbidity, mortality and also tourism potential of an area and decision-making and urban planning (Kozłowska-Szczęsna et al. 2004; Błażejczyk and Kunert 2011; Idzikowska 2011; Bröde et al. 2013; Matzarakis et al. 2014; Bleta et al. 2014; Kuchcik 2017). Bioclimatic conditions in Poland have been studied in various aspects both in larger and smaller urban areas and other attractive tourist places among others (Czarnecka et al. 2011; Kuchcik et al. 2013; Błażejczyk et al. 2014a; Nidzgorska-Lencewicz and Mąkosza 2013; Nidzgorska-Lencewicz 2015; Bartoszek et al. 2017; Mąkosza and Nidzgorska-Lencewicz 2017; Kolendowicz et al. 2018; Rozbicka and Rozbicki 2018a, b; Owczarek 2019; Koźmiński and Michalska 2019).

There are many different indicators describing the impact of the atmospheric environment on the human body. However, most of them are not directly related to physiological reactions of the body that occur in the body in response to the prevailing thermal conditions. In the nineties of the twentieth century, so-called multi-node models of human body heat balance were developed to describe all mechanisms of thermoregulation. One of these models was the basis for the elaboration of the Universal Thermal Load Index—UTCI (Universal Thermal Climate Index), whose purpose is to assess the degree of thermal stress to which the human body is exposed (Fiala et al. 2012). It is assumed that UTCI provides information about thermal and physiological processes in the whole spectrum of possible environmental conditions (including climatic seasonality) and in all spatial scales. The UTCI should be useful in crucial applications in human bioclimatology, such as daily forecasts and extreme weather warnings, bioclimatic mapping, urban and regional planning, and also environmental epidemiology, climate therapy and climate impact studies. It is shown in the research carried out by Kunert (2010), Błażejczyk et al. (2010), Novák (2011), Mateeva (2011), Kuchcik et al. (2013), Okoniewska and Więcław (2013), Błażejczyk et al. (2014a), Lindner (2011), Lindner-Cendrowska (2013), Dobek and Krzyżewska (2015), Araźny et al. (2016), Zare et al. (2018) and Yang et al. (2018).

Special indicators, for example, Tourism Climate Index (TCI) developed by Mieczkowski (1985), are used for a better quantification of bioclimatic conditions, especially in terms of climate tourism potential. The newer index, more commonly used is the Climate-Tourism/Transfer-Information-Scheme taking into account the latest scientific approach based on human biometeorology (Lin and Matzarakis 2008; Zaninovic and Matzarakis 2009; Shiue and Matzarakis 2011; Çalışkan et al. 2012; Brosy et al. 2014; Algeciras and Matzarakis 2016). Unlike the previous indicator, it also includes thermal comfort/stress parameters. CTIS is based on a combination of three groups of factors: thermal aspects (includes comfort, heat and cold stress), aesthetic aspect (includes fog, clouds) and physical ones (includes wind velocity, precipitation, sultriness). A new approach in the assessment of climate tourism is also making specific quantification of climate by the use of a set of climate data or set of climatic data forecasted based on climate simulations for present or future periods, which were carried out, among others, by Matzarakis et al. (2013, 2014).

The area of the Warsaw agglomeration is one of the most attractive regions in Poland for various kinds of tourism (cultural, business and urban tourism), as well as for different forms of recreation and active recreation of the inhabitants. The aim of the study is to assess the temporal and spatial difference of heat stress in man based on long-term data (1980–2016). On the basis of the universal thermal climate index (UTCI) and designated Climate-Tourism-Information-Scheme (CTIS) diagrams, a comprehensive and detailed bioclimate assessment was made for three different areas in terms of development of Warsaw agglomeration. CTIS is based on the integration of information of single meteorological elements (fogginess, windiness, sunshine, cloudiness, precipitations, sultriness and dryness) which are strongly connected with the facets of climate on tourism. The obtained results can be used, among others, as a tool in the long-term adaptation and restructuring of the tourism sector as well as a spatial development tool in urban planning.

## Materials and methods

**Fig. 1:**
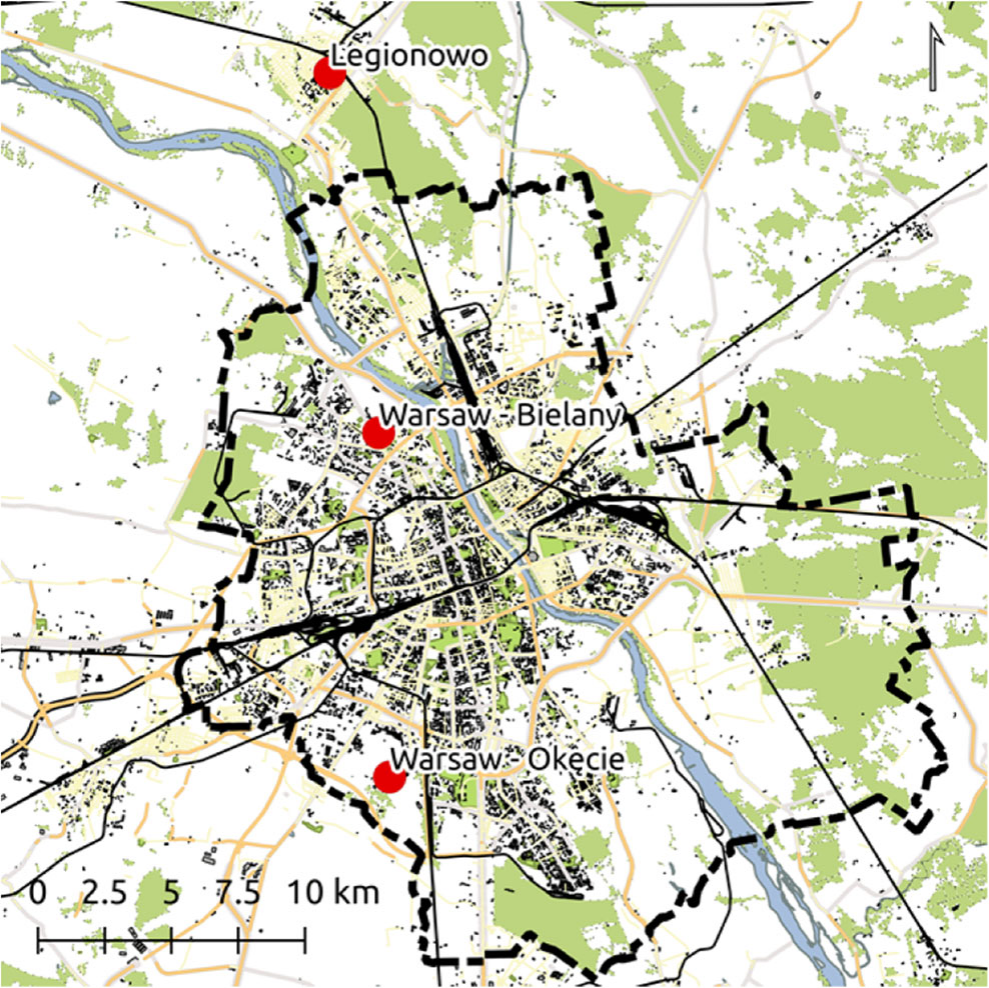
Location of the analysed stations in Warsaw agglomeration

The area of the research in this work is Warsaw agglomeration located in the central-eastern part of Poland in Middle-Mazovian Lowland. It is the largest city in terms of population (over 2.7 million) and also one of the most attractive and most visited by tourists (over 25 million a year; Tourism in Warsaw. Report 2017 (2019) https://issuu.com/visitwarsaw/docs/tourism_in_warsaw_report_2017). Bioclimatic conditions were analysed on the basis of daily values of meteorological elements obtained from IMGW–BIP for three stations: Warsaw-Okęcie, Warsaw-Bielany and Legionowo (Fig. 1) at 12 UTC 1980–2016 (37 years). Three places differentiated in terms of space were selected, which represent different areas of the city. Station Warsaw-Bielany (φ = 52°17′N; λ = 20°58′E; H = 98 m a.s.l.) is located in the north-western part of Warsaw and represents a typical city area with different spatial organisation, types of building, types of urban green and type of land use. Housing estates, service facilities and green areas are close the station. Station Warsaw-Okęcie (φ = 52°10′N; λ = 20°58′E, H = 106 m a.s.l.) is located southwest of Warsaw in the Chopin International Airport area. It represents city outskirt with open area and distant from housing estates. Station Legionowo (φ = 52°24′N; λ = 20°58′E, H = 94 m a.s.l.) is located North of Warsaw and represents suburban area with low building housing estates.

Air temperature (°C), relative humidity (%), wind velocity (m s^−1^) on 10 m a.g.l. and cloudiness (0–8) were used in the work. The Universal Thermal Climate Index (UTCI) and average (*T*_mrt,_ °C) were calculated on the basis of meteorological data by the use of Bioklima 2.6 (2019) (https://www.igipz.pan.pl/Bioklima-zgik.html). Decadal values of bioclimatic indicators are common used as useful and clear information about bioclimatic conditions, especially for tourism.

According to scale of heat stress (Table 1), based on the obtained index values, the body’s thermal stress were assessed. Temporal variability and frequency for thermal stress categories of the UTCI were also determined and the trend lines for the analysed years were determined.

**Table 1:**
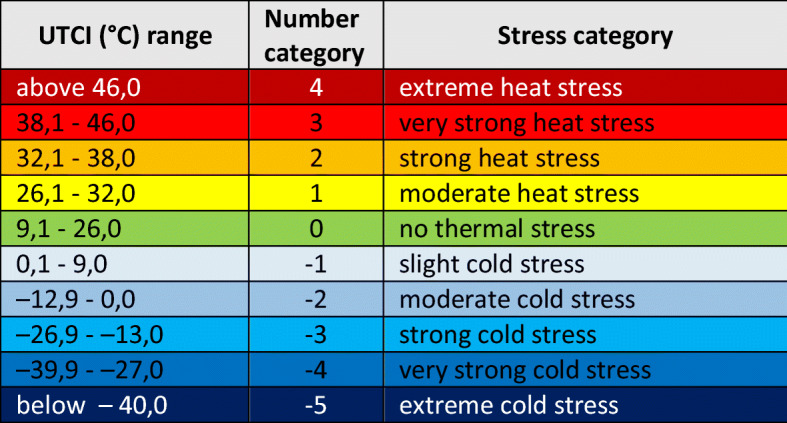
UTCI assessment scale categorised in terms of thermal stress (Fiala et al. 2012)

The Climate-Tourism-Information-Schemes (CTIS) were prepared for analysed stations, which presents the probability of weather component occurrence important for both residents and tourists (Matzarakis et al. 2014). CTIS is a practical and transparent method of graphically presenting parameters related to tourism and provides relative frequency classes and frequencies of extreme weather events on a 10-day (decades) and monthly time scale. A frequency 100% indicates that each day in a month is characterised by the respective condition. The method combines climatological and tourism-related components and simplifies climate information for tourism. (Matzarakis et al. 2013). A new approach based on climate thresholds Climate-Tourism/Transfer-Information-Scheme (CTIS) was applied among others by Matzarakis et al. (2013, 2014), as presented here. The occurrence of different weather characteristic is determined for individual days of the calendar year. They represent the comfort and thermal discomfort of the body and the specific properties of meteorological elements. In this work, thermal comfort and cold stress were determined using the UTCI representing the midday observation data. The other variables represent daily data. After defining the individual CTIS components, the frequency of the various components in the next decades of the year is calculated for each day (Błażejczyk and Kunert 2011). The elements of the climate and tourist information diagram are the following weather features: foggy days (relative humidity > 93%); windy days (wind speed >8 m s^−1^); sunny days (*N* < 5 octas); cloudy days (*N* > 6 octas); sultry days (vapour pressure > 18 hPa); rainy days (daily P > 5 mm), dry days (P < 1 mm), thermal comfort (9 °C > UTCI < 26 °C); hot stress (UTCI > 32 °C); cold stress (UTCI < − 13 °C).

## Results and discussion

**Fig. 2:**
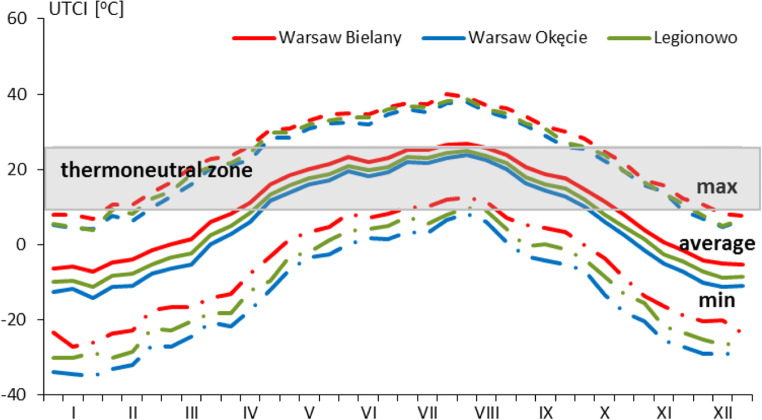
Average, maximal and minimal 10 days of UTCI index at 12 UTC in analysed stations, 1980–2016

The average annual value of the UTCI in the years 1980–2016 in the Warsaw agglomeration was 7.7 °C, which corresponded to the ‘slight cold stress’ and ranged from 5.4 °C at the Okęcie station to 10.4 °C at the Bielany station (Fig. 2). In the annual course, the highest maximum value of the UTCI occurred at the Bielany station in the second decade of July and amounted to 40.1 °C, which corresponded to the ‘very strong heat stress’. The lowest UTCI value, the minimum value, was − 35.1 °C, and it corresponded to the ‘very strong cold stress’ and recorded in the third decade of January at the Okęcie station. In the annual course, the highest average decade values occurred in the third decade of July and the first decade of August and ranged from 23.9 °C (Okęcie) to 26.8 °C (Bielany), which corresponded to the heat categories from ‘no thermal stress’ to ‘moderate heat stress’. The lowest average UTCI values usually occurred in the third decade of January and ranged from − 7.3 °C (Bielany) to − 14.3 °C (Okęcie) and corresponded to ‘moderate cold stress’ and ‘strong cold stress’ (Fig. 2). A similar range of values was obtained by Kuchcik (2017) from 40 years 1975–2014 UTCI in Warsaw, Lindner (2011) for Warsaw in the period 2000–2009 and Mąkosza (2013) on the Lubuskie Voivodeship for the period 1971–2006 obtained slightly lower values of UTCI for summer months and higher for the winter months.

The annual course of average, maximum and minimum 10-day (decades) UTCI values indicates the existence of significant differences in bioclimatic conditions in the studied areas. The greatest differentiation occurs in the average course of UTCI values at the Bielany station compared with other stations. The period of the highest average UTCI values in category 0 ‘no thermal stress’ lasts the longest in this station from the second decade of April to the second decade of October in average. However, at other stations, it is slightly shorter and lasts from the third decade of April to the first decade of October (Fig. 2). During this period, the maximum values of the UTCI reach category 2 ‘strong heat stress’ at all stations most often. The minimum values of the UTCI in this period only at the Bielany station reach both category 0 ‘no thermal stress’ in July and August and category − 1 ‘slight cold stress’. The minimum values of the UTCI indicator in this period at the Bielany station reach both category 0 ‘no thermal stress’ in July and August and category − 1 ‘slight cold stress’. The ‘slight cold stress’ category occurred at other stations most often.

In the transitional periods of the year, i.e. spring and autumn category, − 1 ‘slight cold stress’ occurs most often. These periods are the shortest among all but at the Bielany station last the longer in comparison with other stations—from the first decade of March to the first decade of April for spring and from the third decade of October to the second decade of November for autumn. In the rest of the year (mainly in the winter months), i.e. from the third decade of November to the third decade of February, category − 2 ‘moderate cold stress’ usually occurs for the Bielany station, while at that time both the category ‘strong cold stress’ and ‘moderate cold stress’ dominate at other stations.

**Fig. 3:**
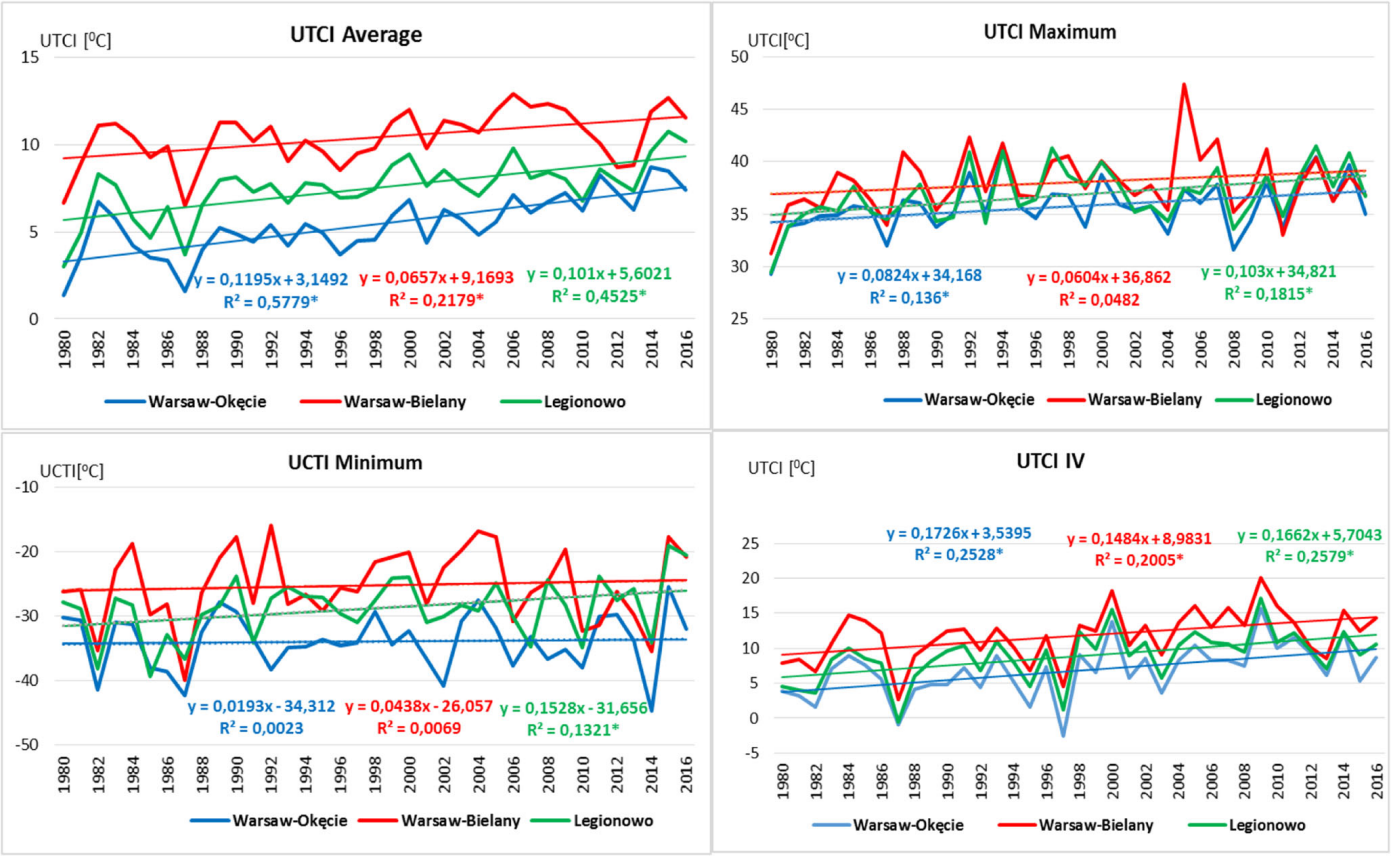
Variability of average, maximum and minimum value of UTCI index at 12 UTC in analysed stations and in April in 1980–2016 with linear trend. *Significant at 0.05

In the next step of the research, an analysis of linear trends of long-term bioclimatic conditions was made. Trends were determined for the average, maximum and minimum UTCI value, for months and the number of days for the thermal heat categories. The aforementioned characteristics were evaluated based on the determined regression equations and determination coefficients (Fig. 3). A statistically significant (*p* < 0.05) positive linear trend of the average value was observed at all analysed stations of the Warsaw agglomeration. A positive linear trend was also obtained for the maximum and minimum values, but they are statistically significant only in case of Okęcie and Legionowo for the maximum value and only for the Legionowo in case of the minimum value. Trends for all months were also analysed, but because of their considerable size, only results for one sample month of April were included in the paper (Fig. 3). A positive linear trend was obtained for all months, but statistically significant for all analysed areas, i.e. for all three stations only in the case of 4 months: April (Fig. 3), June, July, August and also in case of September, November and December for the stations Bielany and Legionowo. Mąkosza (2013) for the area of Lubuskie Voivodeship in the period 1971–2006 obtained similar results although for some months obtained, the trend was negative (September, February). For Warsaw agglomeration, there was not such a case.

**Fig. 4:**
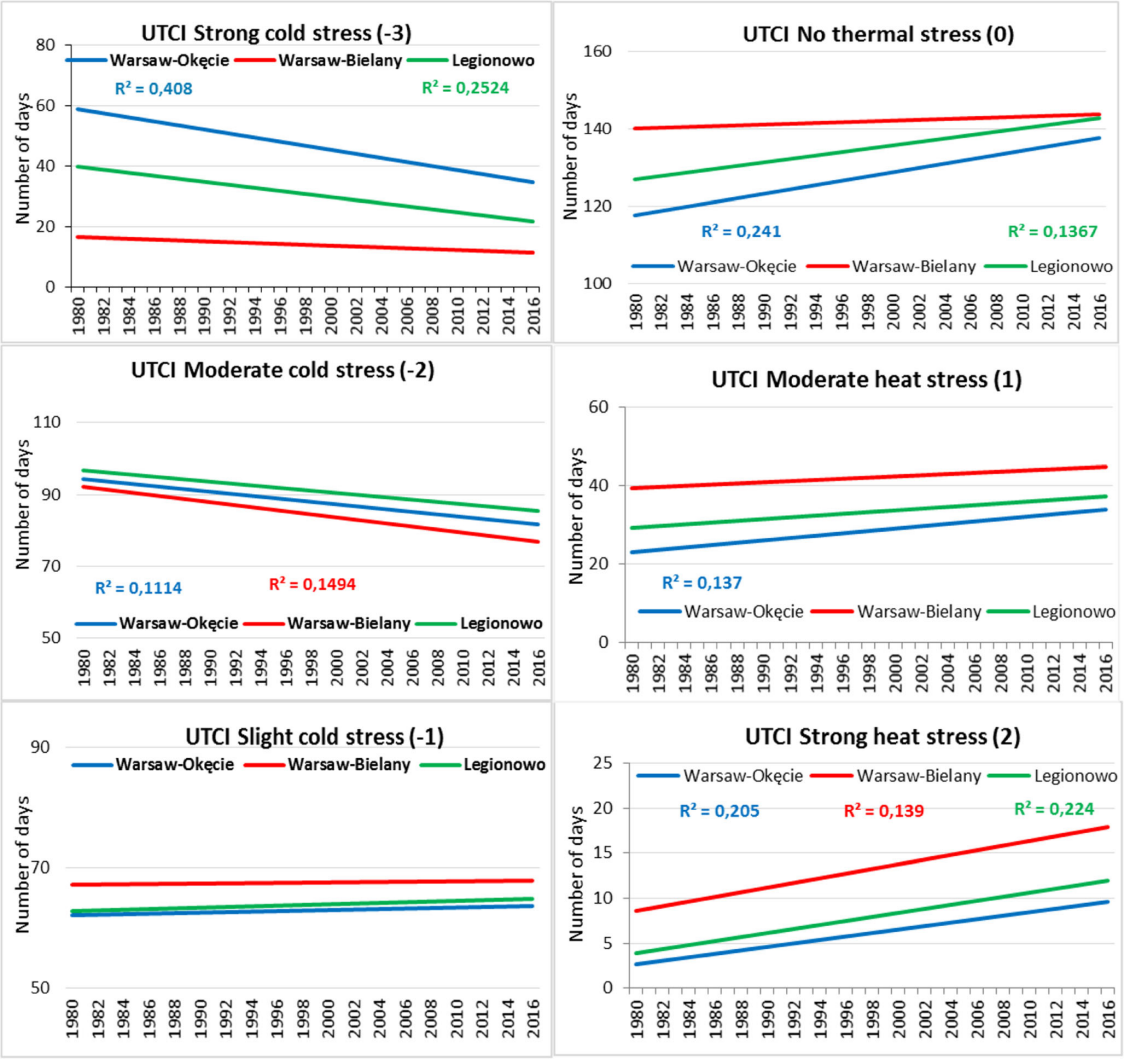
Average number of days with thermal stress categories according to UTCI in the succeeding years of 1980–2016 in analysed stations with trend lines. *The coefficients of determination shown are significant at a statistical level of 0.05

In the next stage, a linear trend of the number of days for individual thermal stress categories was determined (Fig. 4). A negative linear trend of the number of days was observed for thermal stress categories related to cold stress categories (− 3, − 2 or ‘strong and moderate cold stress’). For the ‘strong cold stress’, the trend was statistically significant (*p* < 0.05) only in case of Okęcie and Legionowo stations, and for the ‘moderate cold stress’ only in case of Okęcie and Bielany stations. A positive linear trend was observed for all analysed thermal stress categories, but statistically significant in case of all three stations only for category 2 ‘strong heat stress’. Among determined trends of the number of days with heat stress for all analysed stations, the most frequent ones statistically significantly occur in case of Okęcie and the least frequent in case of Bielany (only for two categories). Mąkosza (2013) in her research from 1971 to 2006 for Lubuskie Voivodeship also obtained a positive trend for the categories ‘no thermal stress’ and categories related to heat stress (UTCI > 32 °C) and a negative trend for UTCI < − 13 °C (category related to cold stress). Półrolniczak et al. (2016) and Kolendowicz et al. (2018) for the coastal towns of the Baltic Sea recorded similar results of the trend analysis.

**Fig. 5:**
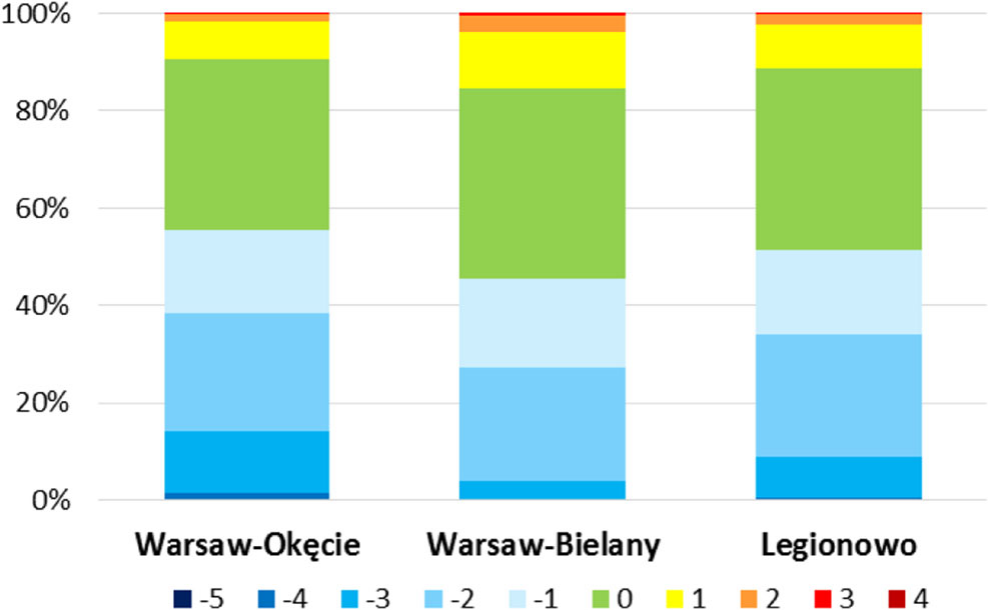
Frequency (%) of thermal stress categories according to UTCI index at 12 UTC in period 1980–2016 in analysed stations

**Fig. 6:**
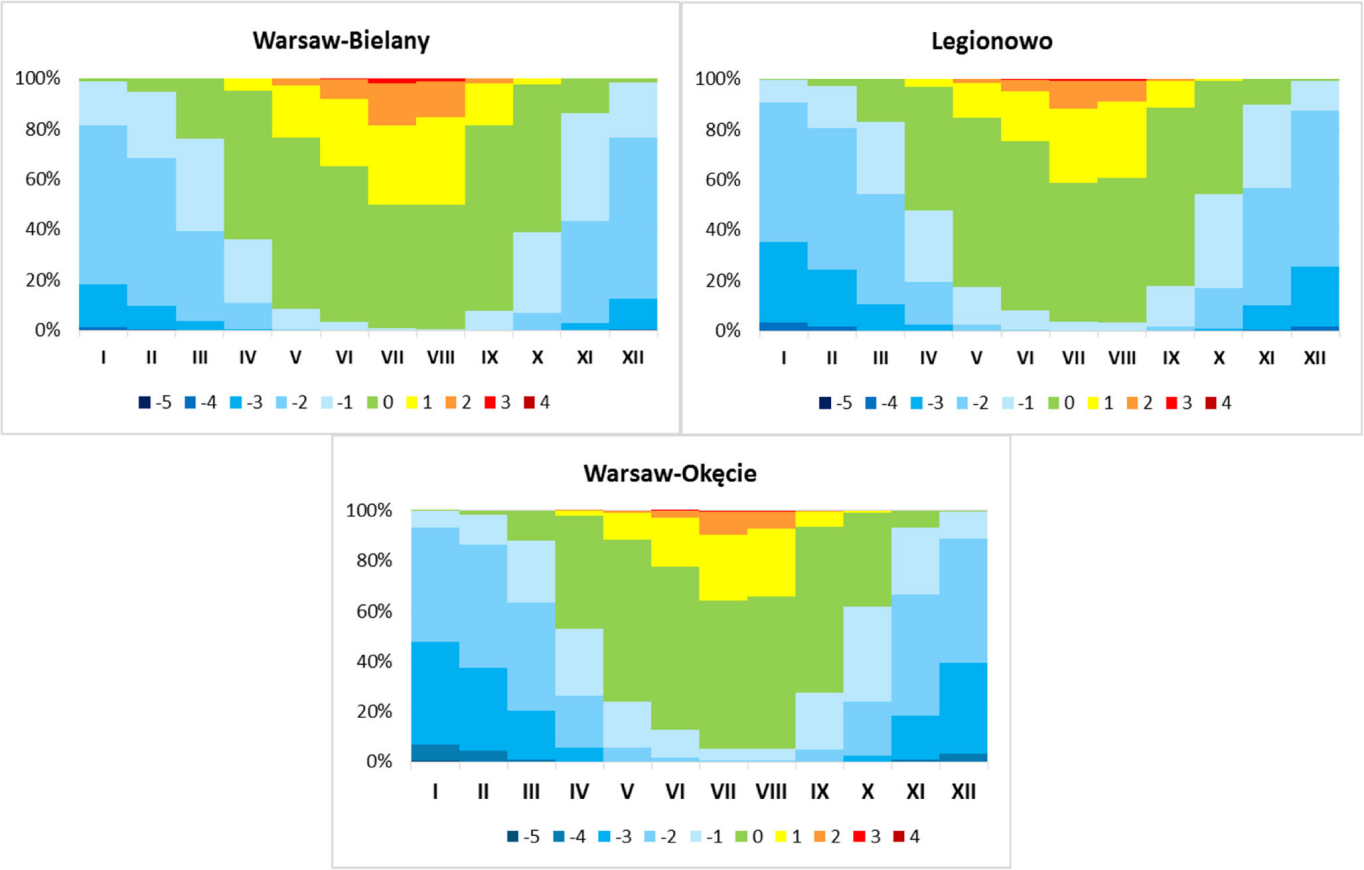
Frequency (%) of thermal stress categories by UTCI index at 12 UTC in particular months in analysed stations, 1980–2016

**Fig. 7:**
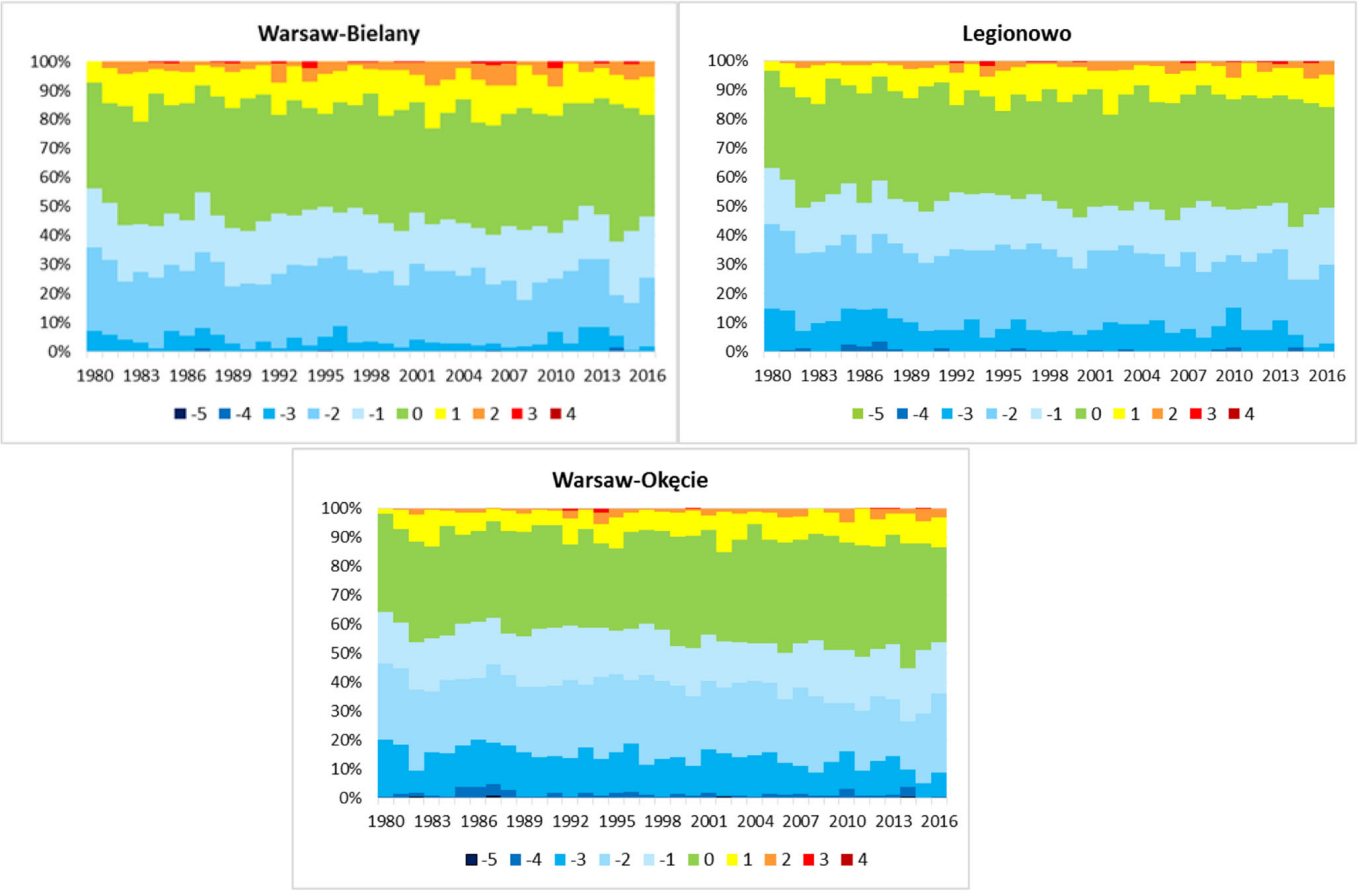
Frequency (%) of thermal stress categories according to UTCI index at 12 UTC in particular years in analysed stations, 1980–2016

The next stage of the work was determining the frequency of the UTCI value in relation to the category of heat stress assessment (Figs. 5, 6, and 7). The most frequent thermal stress category for the UTCI in all period 1980–2016 was ‘no heat stress’ which varies from 34.9% (in case of Okęcie) to 38.9% (Bielany) (Fig. 5). Similar results were obtained by Kolendowicz et al. (2018) for the cities of the Baltic Coast from the period 1981–2014 and Lindner (2011) for the Okęcie station from the period 2000–2009. Warsaw is characterised by significant occurrence of the burdensome conditions linked with cold stress—the most from category (− 2) of ‘moderate cold stress’ (23–25% during the year) and slightly less with category (− 1) ‘slight cold stress’ (17–18%). Days with ‘strong and very strong cold stress’—categories − 3 and − 4—occur less frequently and with varying frequencies at studied stations. Their predominant frequencies are at the Okęcie and Legionowo stations, where they occur on 14% and 9% days, respectively, while at the Bielany station they occur only 4%. Days with the − 4 category ‘very strong cold stress’ most often appeared at the Okęcie station (1.3%), while at other stations the frequency was 0.2–0.6%. ‘Extreme cold stress’ conditions appeared very rarely in the examined stations. During the analysed years 1980–2016, eight such cases were recorded, including six cases at the Okęcie station (0.04%).

In the categories of heat stress, most often there were days from category 1 ‘moderate heat stress’ and the frequency ranged from 8% to 12% for Okęcie and Bielany stations, respectively. Days from category 2 ‘strong heat stress’ occurred less frequently with the frequency from about 2% for Okęcie and Legionowo to 3.6% at the Bielany station. Days with category 3 ‘very strong heat stress’ occurred relatively rarely in 45 cases at Bielany station (0.3%), 22 cases in Legionowo (0.2%) and the least in 13 cases at Okęcie (0.1%). Days from category 4 ‘extreme heat stress’ were sporadically recorded in a total of four cases, including two at Bielany station (0.015%) (Fig. 5). For these categories, the results obtained by Kuchcik (2017) in Warsaw (1975–2014) were similar.

Significant UTCI differences between the Bielany (city centre) station and the other Okęcie (suburban) and Legionowo (outskirts) stations can be clearly observed if comparing monthly data (Fig. 6). Category 0 ‘no thermal stress’ is dominant among other categories from April to October, and it ranges from approximately 38% in October (Okęcie) to about 74% in September (Bielany). ‘No thermal stress’ occurred above 60% at each station from May to April. Heat stress from ‘moderate heat stress’ to ‘extreme heat stress’ occurred around 50% in the city centre (Bielany), while at outskirt stations about 40%. At Bielany station, the frequency of strong and very strong stress was 18.3% whereas at other stations it ranged between 9% and 12%. ‘Extreme heat stress’ was recorded only at Bielany station in July with frequency 0.1%. Thermal stress categories related to cold stress, i.e. category − 1 ‘slight cold stress’ and category − 2 ‘moderate cold stress’, are dominant at Okęcie from October to March. In amount, they have the frequency approximately 48% (in January), and at other stations they are 35% in Legionowo and 18% for Bielany, respectively.

The frequency of thermal stress determined on the basis of the UTCI shows a large variation in particular years, as well as different areas (Fig. 7). The category 0 ‘no thermal stress’ was the most frequent. It ranged from 30% (years 1992, 1995) to 50% (2014) with the highest frequency in Bielany. Cold stress (categories from − 1 to − 5) ranged between 9% and 11%. Occurrence of higher frequencies of these categories clearly shows spatial diversity and the impact of the city between its outskirts (Okęcie) and suburbs (Legionowo) than in its area (Bielany). Category − 5 ‘extreme cold stress’ occurred only in Okęcie station with frequency of approximately 0.5% (1982, 1987, 2014). Frequency of categories related to the heat stress (1–4) ranged between 2% and 4%. In the case of these categories, the highest values occurred in Bielany station in comparison with other areas. The category ‘extreme heat stress’ (4) was only noticed (0.3%) at Bielany where it occurred only once in 2005. Higher frequency of thermal stress related to cold stress was observed in the early 1980s (mainly in Okęcie) and heat stress at the beginning of the twenty-first century (mainly in Bielany).

**Fig. 8:**
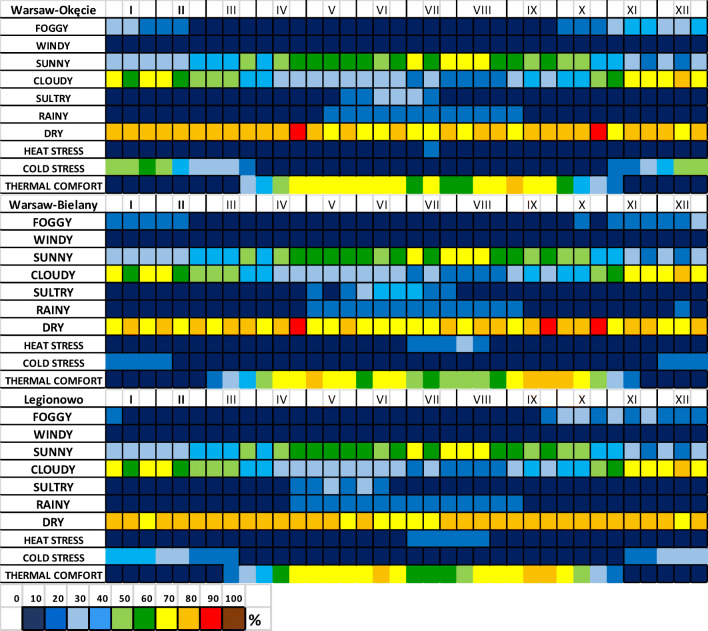
The Climate-Tourism-Information-Scheme (CTIS) for analysed stations (frequency of occurrence in the period 1980–2016 in percent). The colour of each blank represents each of the parameters for specific criteria during 10-day periods

The last stage of the study was intended to determine the Climate-Tourist-Information-Scheme (CTIS), which is a simple and clear information about the variability of bioclimatic conditions mainly for tourism, but also for other various forms of recreation and human activity (Fig. 8). Cloudy days are the biggest limitation during the year for tourism and other forms of recreation. They occur mainly in winter with a frequency above 50% in the whole area of Warsaw agglomeration. The highest frequency of cloudy days occurred from the second decade of November to the first decade of February. The analysis of the diagrams shows a greater spatial diversity between the areas in case of thermal components analysed, which were determined using UTCI. An unfavourable feature is the cold stress load occurring in winter. This situation takes place at the Okęcie station most often with approximately 50% of probability mainly in the period from the second decade of December to the first decade of February with the greatest intensity in the third decade of January. At the other stations, the frequency of this thermal stress does not exceed 40% at Legionowo, and at the Bielany station less than 20% (Fig. 8).

For summer months (especially July and August), the main limitation may be heat stress. The largest occurs at Bielany station; however, it does not exceed 20% and in the case of other stations—10%. The second limitation in the summer is the number of sultry days. It reaches almost 40% at the Bielany station in the third decade of June.

The most favourable conditions in terms of thermal comfort occur in spring and autumn, reaching over 70% probability especially in the first decade of May and in September to the first decade of October particularly at the Bielany station. Other components do not significantly affect the planning of recreation and activity; they occur rarely and with a low frequency. For example, rainy days occur mainly in summer (July) and do not exceed 20%, foggy days occurring mainly in winter and autumn reaching 30% (at Okęcie) and about 20% (at Bielany and Legionowo).

Bioclimatic research on heat stress in Warsaw over the last 40 years has been carried out by Kuchcik (2017) for the period 1975–2014 and in this paper for a similar period (1980–2016). UTCI has been published comprehensively in 2012 in a special issue of the *International Journal of Biometeorology*. Only several research studies have appeared since then. Although in recent years there are more and more of them, still not many of them concern the use of UTCI to assess thermal stress in urban areas. Błażejczyk and Kunert (2010) compared heat stress in 15 European cities and in the summer and winter months. They found significantly increased heat stress in larger conurbations especially in southern Europe. There is still little research on the spatial diversity of biometeorological conditions in urban areas, but those that have been done are concerned the area being analysed. Błażejczyk et al. (2014a) showed the spatial diversity of biothermal conditions in the centre of Warsaw and in outskirts as well as showed a strong impact on the body’s thermal stress of individual types of buildings and land use and a significant impact of urban green especially high (trees) on alleviating heat stress in Warsaw. Błażejczyk et al. (2014b), for the period 2005–2010, studied the spatial variability of UTCI at the local scale using urban areas with different sizes and geographical locations. The research on urban heat stress was conducted in urban and rural areas in Warsaw.

They obtained the UTCI that is very sensitive to even small changes in meteorological variables induced by different urban structures at very detailed scale. An important role of biologically active surface on mitigation of biothermal conditions was also found. Low density estates with a large proportion of biologically vital surfaces and trees can create relatively mild biothermal conditions (Kuchcik et al. 2019). Additional advantage of UTCI is the opportunity of application to GIS tools which can give a very clear presentation of the spatial diversity of heat stress. City centres and industrial districts are clearly seen as the areas with the greatest heat stress. Cartographic presentations of the UTCI distribution were made by Błażejczyk and Kunert (2011) for Warsaw as well as by Milewski (2013) for the mountain region of Ziemia Kłodzka in the Polish Sudety Mountains. Kuchcik (2017) comprehensively presented thermal and bioclimatic conditions in 12 cities in Poland, among others in Warsaw for the period 1975–2014, and their impact on mortality. The results show for most cities from 1975 to 1994 a clear but mild increase in the frequency of heat stress load by 0.2–0.6 days/year and a clearly decreasing number of days with severe cold stress by 0.8–3.0 days/year usually from the nineties of the twentieth century. Similar results were obtained among by Mąkosza (2013), Dobek and Krzyżewska (2015), Półrolniczak et al. (2016), Araźny et al. (2016), Kolendowicz et al. (2018) and Koźmiński and Michalska (2019) for different Polish towns.

In turn, in the case of the research on the relationship between UTCI and mortality, Kuchcik (2017) found that if the heat stress was very severe, the risk of death was elevated by more than 20% and even more than 30% in the case of Warsaw. In the case of adaptation to extreme thermal conditions, Kuchcik (2017) has shown that Poles have adapted to hot conditions, especially women; men have adapted to a slightly lesser extent. Adaptation to extremely cold conditions was not visible.

In this study, as one of the methods of assessing the comprehensive weather for the needs of tourism, the Climate-Tourism-Information-Scheme (CTIS) diagram was used. The number of similar publications for Polish conditions is negligible. This is surprising, especially because the CTIS diagram is quite common and is the frequently used method in assessing tourism needs in Europe and the world. Only two studies present the results of this method for Poland. The first comprehensive monograph was elaborated by Błażejczyk and Kunert (2011) for the period 1971–1990, in which CTIS diagrams were developed for different regions of Poland, among others for Warsaw. The main difference in comparison with the results of this paper is the occurrence of milder bioclimatic conditions, especially in summer. Similar results to the ones that were obtained in this study were presented in the other paper by Lindner-Cendrowska and Błażejczyk (2014) for Warsaw from the period 2003–2012. They stated that bioclimatic conditions are the biggest limitation for outdoor recreation and urban tourism. These are cloudy days occurring above 50% in the winter and days with heat stress occurring even 20% at city stations in the summer.

## Conclusions

The results of the research carried out on an urban area show very good applicability of the Universal Thermal Climate Index in studies of heat stress caused by different structures in the city.

UTCI very clearly shows the general features of the urban bioclimate compared with the suburban and outskirts in the annual course. The most intensive and the most frequent heat stress inside the city than in the suburbs and outskirts was proved.

Trend gives the opportunity of forecasting how the frequency of occurrence in individual thermal thresholds will change in the future. A significantly higher increase of the frequency in heat stress categories can be expected inside a city (Bielany) to 60% than a suburb (40%) and outskirts (43%) in 2050.

Assuming the decreasing trend of the frequency of days with strong cold stress is maintained, this frequency will decrease to 1% in the mid-50s for Legionowo, in the mid-60s for Okęcie and at the beginning of the seventies of twenty-first century at the Bielany station.

The Climate-Tourism-Information-Scheme (CTIS) findings obtained were used to prepare a simple and clear bioclimate and tourism climate information that can be understood by everyone.

There is a significant need to carry out detailed research that comprehensively covers weather conditions on the bioclimatic conditions of tourism and other forms of recreation in Poland, particularly in areas of the cities where a significant part of tourism is concentrated.
